# Hypercholesterolemia negatively influences morphology and molecular markers of epithelial cells within the choroid plexus in rabbits

**DOI:** 10.1186/s12987-020-0175-0

**Published:** 2020-02-04

**Authors:** Fumiko Obata, Keishi Narita

**Affiliations:** 1 Department of Molecular Pathology, Faculty of Medicine, University of Yamanashi, 1110 Shimokato, Chuo, Yamanashi 409-3898 Japan; 2 Department of Anatomy and Cell Biology, Faculty of Medicine, University of Yamanashi, 1110 Shimokato, Chuo, Yamanashi 409-3898 Japan

**Keywords:** Choroid plexus epithelial cell, Hyperlipidemia, Rabbit

## Abstract

**Background:**

Choroid plexus (CP) is an important tissue not only to produce cerebrospinal fluid (CSF) but also to regulate substances that are secreted into or absorbed from CSF through blood–cerebrospinal fluid barrier (BCSFB) formed by CP epithelial cells (CPECs). CPECs display signs of deterioration in aged and diseased people. However, whether CPECs in hypercholesterolemic animals develop such damage is not known.

**Methods:**

We used cholesterol-fed wild-type or Watanabe hereditary hyperlipidemic (WHHL) rabbits of identical age to determine CPEC changes in terms of morphology and protein expression/localization.

**Results:**

Compared with non-cholesterol-fed control rabbits, prolonged exposure to cholesterol reduced CPEC height and increased lipofuscin levels in CPECs, indicating cellular damage. Expression of aquaporin 1 on the apical membranes of CPECs was diminished in cholesterol-exposed rabbits, implying a reduced CSF-producing function in the CP. The rabbit macrophage-specific antibody (RAM11) immunoreaction became positive in CPECs adjacent to foam cells, indicating an alteration in this cell type.

**Conclusion:**

Cholesterol insults from the circulation (which is reflected by foam-cell accumulation in the CP) induce CPEC dysfunction, and the latter seems to be enhanced by foam cells in hypercholesterolemic rabbits.

## Background

The choroid plexus (CP) is a secretory and scavenging tissue in ventricles of the brain. It has important functions in brain development and homeostasis [1, 2]. Choroid plexus epithelial cells (CPECs) have the major role in the CP functions. While producing the cerebrospinal fluid (CSF) into the brain ventricles, CPECs form a tight junction known as the blood–cerebrospinal fluid barrier (BCSFB) to allow only selective substances (ions, amino acids, folate, glucose, transthyretin, vitamins B6, B12, C and E) to traffic between the systemic circulation and the CSF [1–4]. CPECs also function as “entry gates” of leukocyte passage into CSF [5–8]. In addition to transepithelial transport, CPECs also synthesize and secrete biologically active molecules into CSF. Recent studies have revealed that CPECs secrete exosomes carrying microRNAs [9]. Furthermore, CPECs transport proteins such as WNT5A via lipoproteins as vehicles into CSF [10, 11].

Several scholars have demonstrated that CPEC impairment influences brain function. For example, hydrocephalus occurs due to CP hyperplasia [12–14] and folate deficiency-associated neurologic disorders occur due to a reduced transfer of folate through CPECs with gene mutations in the proton-coupled folate transporter or folate receptor-α [14]. Several alterations occur in CPECs upon aging. Changes such as a reduction in the height of CPECs, accumulation of lipofuscin and a decrease in the rate of CSF production are markers of CPEC impairment [15–18]. These changes are enhanced further in Alzheimer’s disease (AD) patients [16, 19, 20].

Epidemiologic studies suggest that high levels of cholesterol in blood during middle age are a risk factor for AD in later life [21]. Correspondingly, cholesterol-fed rabbits with hyperlipidemia, and spontaneously hyperlipidemic, Watanabe hereditary hyperlipidemic (WHHL) rabbits exhibit pathologic changes similar to AD and have been regarded as valid AD models [22–24].

To study CPEC defects in relation to cholesterol accumulation within the CP, we used hypercholesterolemic rabbit models in this study. Unlike rodents, rabbits are cholesterol diet sensitive and have lipid metabolism similar to human [25, 26]. In fact, accumulation of foam cells (FCs) in the CP of hyperlipidemic rabbits has been reported by Chen et al. [27]. The authors demonstrated detection of CP tissue by magnetic resonance imaging and FCs by histology. However, damage to CPECs in hyperlipidemic rabbits has not been investigated previously. To test the hypothesis that cholesterol insults from the circulation induce CPEC deterioration, we assessed several markers of CPEC in hyperlipidemic rabbits compared with age-matched normal rabbits.

## Methods

### Animals

Male Japanese White (JW) rabbits (15 weeks) were purchased from Japan SLC (Hamamatsu, Japan). JW rabbits were divided into two groups randomly. The first group (n = 4) consumed a standard chow (0% cholesterol) diet containing 17.65% protein, 3.50% fat and 14.77% fiber (CLEA Japan, Tokyo, Japan). The second group (n = 6) consumed a 0.3%-cholesterol diet with 3% soybean oil supplemented with the standard chow diet. In this second group, a model of diet-induced hypercholesterolemia (dHC) was created. A third group consisted of spontaneously hyperlipidemic WHHL rabbits (31 weeks; n = 7), which were a kind gift from Dr. Shiomi (Kobe University, Kobe, Japan).

JW rabbits were fed the assigned diets for 16 weeks. Rabbits were habituated for 1 week with the standard chow diet before experiments. Food and water were given ad libitum. At the time of sacrifice, rabbits in all three groups were 32 weeks of age. Pig heads used to isolate the CP were obtained from a local slaughterhouse. Animal experiments were approved by the Institutional Animal Care Committee of the University of Yamanashi (Kofu, Japan).

### Plasma lipid isolation and total cholesterol measurement

Before sacrificing animals, 1.5 mL blood from ear artery was taken to microtubes containing 15 μL of 0.5 M ethylenediaminetetraacetic acid (EDTA) pH 8.0 and 15 μL (0.1 trypsin inhibitor unit) of aprotinin (A6279, Sigma-Aldrich Japan, Tokyo, Japan). EDTA-treated blood was centrifuged at 4000 rpm for 20 min at 4 °C and plasma was isolated. Total cholesterol was measured with cholesterol E-test Wako (Wako Pure Chemical Corporation, Osaka, Japan).

### Tissue processing

At the end of diet feeding for groups 1 and 2 or after the habituation period for group 3, animals were sacrificed by intravenous injection (64.8 mg/kg bodyweight) of pentobarbital sodium (Somnopentyl™; Kyoritsu Seiyaku, Tokyo, Japan). Brains were fixed in 10% neutralized formalin at 4 °C for 1 week. Brains were trimmed into 5 mm-thick coronal pieces and processed for paraffin sections. Blocks which included the CP in the lateral ventricles (LVs) and third ventricle (3 V) were chosen for further sectioning.

### Measurement of CPEC height

Paraffin sections of thickness 3 µm were made and stained with hematoxylin and eosin. Using a light microscope (BX53; Olympus, Tokyo, Japan) with a 40× objective lens, fields were viewed and CPEC height measured using WinROOF (Mitani, Tokyo, Japan). FCs were recognized as cells within stroma of the CP with fine pink-colored punctuations in the cytoplasm and small-sized eccentric nucleus [28]. Approximately 50 CPECs with or without contact with FCs were measured per rabbit.

### Lipofuscin measurement

Sections used for measurement of CPEC height were observed with a confocal microscope (FV1000; Olympus). Lipofuscin emits broad-spectrum autofluorescence, so 4′,6-diamidino-2-phenylindole, fluorescein isothiocyanate, tetramethylrhodamine, and Cy5 channels were observed to confirm lipofuscin signals [29]. Images of 100× objective fields were taken within the fluorescein-isothiocyanate channel [30]. A square region of interest (RoI) that fitted within the CPEC cytoplasm was used to measure white pixels (positive signals) above a threshold. Ten RoI measurements per image were taken for three images per rabbit. The sum of positive signals from each rabbit was averaged within groups.

### Immunohistochemistry

All procedures were undertaken at room temperature unless stated otherwise. For immunohistochemical staining of formalin-fixed paraffin sections, slides were deparaffinized by incubation in xylene for 7 min (repeated thrice) and dipped subsequently in 100% ethanol for 1 min (repeated twice) and 99.5% ethanol for 1 min (repeated twice). Slides were immersed in 0.3% H_2_O_2_/methanol for 30 min to quench endogenous peroxidase. After endogenous peroxidase had been blocked, slides were washed thrice by 0.01 M phosphate-buffered saline (PBS). Antigens were retrieved using 0.01 M citrate buffer (pH 6.0) with an autoclave for 10 min at 120 °C. After cooling to room temperature, samples were washed thrice with PBS. The primary antibodies applied were anti-aquaporin 1 (AQP1; rabbit polyclonal antibody; diluted 1:2000 with PBS; catalog number, AB3065; Merck, Darmstadt, Germany) and anti-RAM11 antibody (mouse monoclonal; diluted 1:400 (0.09 μg/mL) with PBS; M0633; Dako, Glostrup, Denmark). Antibodies were applied to the sections and incubated overnight in a humidified chamber at 4 °C. Slides were washed with PBS for 5 min (repeated thrice). The secondary antibodies anti-rabbit (Fab’)-peroxidase conjugate [MAX-PO (R); 424142, Nichirei Biosciences, Tokyo, Japan] and anti-mouse (Fab’)-peroxidase conjugate [MAX-PO (M); 424134, Nichirei Biosciences] were applied for 1 h, respectively, in a humidified chamber. Slides were washed with PBS for 5 min (repeated thrice) and incubated with 3-amino-9-ethylcarbazole solution (415011; Nichirei Biosciences) and the reaction stopped with distilled water. Samples were washed with running tap-water for 5 min. Nuclei were stained with hematoxylin for 3 s and washed with running tap-water for 5 min, and then washed with distilled water for another 5 min (repeated thrice). Slides were covered with aqueous mounting medium (Aquatex™; 1.08562.0050; Merck).

### Porcine CPEC culture

Primary CPECs were prepared as described previously [31]. Cells grown to high confluency in a coated 10-cm culture dish were trypsinized and passaged on Transwell™ inserts (3413; Costar, Corning, NY, USA) precoated with Matrigel™ (356234; BD Biosciences, San Jose, CA, USA) at 5.8 × 10^5^ cells/cm^2^.

### Quantitative reverse transcription-polymerase chain reaction (qRT-PCR)

Primary CPECs were incubated for 1–2 days in Transwell units. CPECs were rinsed thrice gently with Dulbecco’s modified Eagle’s medium/F12 (11330-032; Thermo Scientific, Waltham, MA, USA) for serum starvation overnight. Then, they were stimulated with 50% fetal bovine serum in Dulbecco’s modified Eagle’s medium/F12 and added to the basal chamber. Cells were incubated for 6 h or 24 h and then lysed in TRIzol^®^ Reagent (15596018; Thermo Scientific) to extract total RNA. RNA samples were reverse-transcribed using a High Capacity cDNA Reverse Transcription kit (4368814; Thermo Scientific). Changes in expression of C-C chemokine ligand 2 (Ccl2) were analyzed by the comparative C_T_ method [32] using Thunderbird™ SYBR qPCR Mix (QPS-201; Toyobo, Tokyo, Japan) and the StepOnePlus™ Real-time PCR system (Thermo Scientific).

The oligonucleotide primers (forward and reverse, respectively) for quantitative PCR were: 5′-ACAGAAGAGTCACCAGCAGCAA-3′ and 5′-GCCCGCGATGGTCTTG-3′ for Ccl2; 5′-GTGTGAACAAATGCAGCATCAA-3′ and 5′-GAGCTGCAGAGGGATCATCTTG-3′ for chemokine C-X3-C motif ligand 1 (Cx3cl1); 5′-GTGCGCCCTTTGCAGTCT-3′ and 5′-GCTTGCTGTAGGAACGGTTCTG-3′ for macrophage migration inhibitory factor (Mif); 5′-GGAAGAACACAGCCAGTGTGAAT-3′ and 5′-TGGCTTCACGGCACTCTCT-3′ for vascular endothelial growth factor-B (Vegfb); 5′-TCCGCCCCAGATTGAAATT-3′ and 5′-TGCTCCGCGTTCATCTTCT-3′ for beta-2-microglobulin (B2m).

Expression was assessed by the comparative C_T_ method. The − ∆∆CT values were calculated using *B2m* as an endogenous reference and time zero as a calibrator.

### Statistical analyses

Statistical analyses were undertaken using SPSS v21 (IBM, Armonk, NY, USA). For a three-sample comparison, one-way analysis of variance was applied if samples had a normal distribution (parametric test). As post hoc tests, the Tukey test was used if an equal variance was assumed, and the Tamhane test was used if an unequal variance was detected by the Levene test. In the case of a non-normal distribution, a nonparametric Kruskal–Wallis test was employed with the Dunn test as a post hoc test. For comparison of two samples, the Mann–Whitney test was used for a non-parametric test and Student’s *t*-test was used for a parametric test. *p *< 0.05 was considered significant. Data are the mean ± standard deviation.

## Results

To determine pathologic changes in CPECs by exposure to high cholesterol levels in plasma, we used two rabbit models of hypercholesterolemia with different degrees of cholesterol exposure. One model was dHC of wild-type rabbits. For creation of this model, JW rabbits were fed a 0.3%-cholesterol diet from 16 to 32 weeks of age [33, 34]. The other model was spontaneous hypercholesterolemia of 32-week-old WHHL rabbits. This spontaneously hyperlipidemic strain carries a genetic mutation in the low-density lipoprotein receptor (LDLR) that slows down the transport of the LDLR to the hepatocyte surface. As a result, lipoprotein absorption in the liver from plasma is reduced due to the low abundance of the LDLR. Thus, WHHL rabbits develop hyperlipidemia from the beginning of their lives, and their age represents the duration of hyperlipidemia [35]. As a control for both models, 32-week-old JW rabbits fed with standard chow (cholesterol exposure for 0 weeks) were used. At the time of sacrifice, plasma total cholesterol (mean ± SD) of control, dHC and WHHL32w were 16.73 ± 2.58, 850.72 ± 696.26 and 893.75 ± 148.19 mg/dL, respectively. We examined CP pathology as: (i) changes in CPEC height (ii) lipofuscin accumulation; (iii) AQP-1 expression; (iv) RAM11 immunoreactivity.

### Reduction in CPEC height

**Fig. 1:**
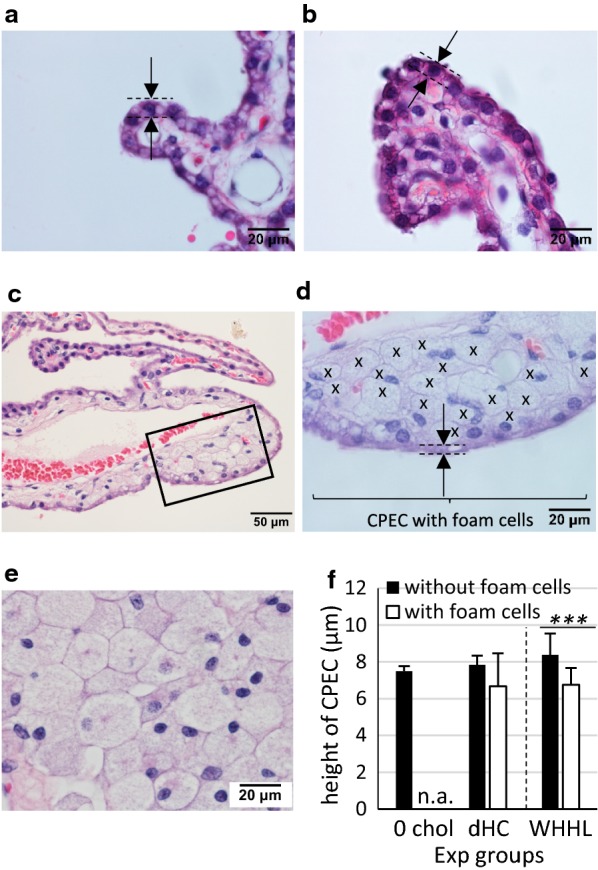
The height of CPECs in contact with foam cells decreases. Representative H&E-stained LV CPs from **a** 0 and **b** 16 weeks of a cholesterol diet (dHC) and **c**–**e** 32-week-old WHHL rabbits. **a**, **b** Show CPECs without contacting foam cells, whereas **c** shows both CPECs that are with (lower 2/3) and without (upper 1/3) FCs. An inset in **c** is shown in **d** with FC marked with (x). The distance between dashed lines (arrows) shown in **a**, **b**, **d** is measured as the CPEC height. In **e**, an example image of clustered FCs with their characteristics of fine pink punctuations in the cytoplasm and small eccentric nuclei is shown. All rabbits were 32 weeks of age. Scale bars in **a**, **b**, **d**, **e** are 20 μm, and a bar in **c** is 50 μm. **f** The mean heights from the measurement are shown (mean ± standard deviation). Bar graphs at the left of the dashed line denote Japanese White rabbits, whereas bars at the right are WHHL rabbits. For CPEC height without foam cells, 0 chol (n = 4) dHC (n = 6) and WHHL (n = 7) are used. For CPEC with foam cells, 0 chol did not have any foam cell mass (not applicable, n.a.), dHC (n = 2, due to foam cell mass detection in 2 out of 6 rabbits) and WHHL (n = 7). ***p < 0.001

In dHC and WHHL rabbit models, cholesterol-exposed CP accumulated FCs within the stroma, and WHHL rabbits had FC mass more frequently than dHC rabbits (Fig. 1a–c). In the WHHL model, formation of cholesterol crystals in the stroma was also observed. In contrast, FC accumulation was not observed in the control group. FC accumulation in the stroma seemed to be associated with the morphologic changes of nearby CPECs. In WHHL rabbits, the height of CPECs contacting FCs (6.76 ± 0.91 μm, n = 7) was significantly different compared with the height of CPECS without FCs (8.38 ± 1.16 μm, n = 7) (values are mean ± SD) (*p *< 0.001) (Fig. 1f, WHHL white vs. black bars). In dHC rabbits, in which 2 out 6 animals had FCs in the CP, difference in height of CPECs with or without FCs cannot be determined statistically (Fig. 1f, dHC white vs. black bars). However the frequency of FC appearance in dHC compared to WHHL suggested that longer cholesterol exposure ensures FC development in the CP. Among CPECs without contact with FCs, the height of cells showed no significant difference (dHC without FCs, 7.83 ± 0.51 μm, n = 6) (*p *= 0.284) (Fig. 1f, black bars dHC vs. WHHL). These data suggested that a longer duration and exposure to a higher concentration of cholesterol induced FC accumulation in CP stroma, which reduced the height of CPECs.

### Lipofuscin accumulation in CPECs

Lipofuscin is an oxidized lipid-containing residue of lysosomal digestion. It has broad autofluorescence [29, 36] and can be used for fluorescence-based detection of cumulative oxidative stress [30]. An increase in lipofuscin levels within CPECs has been reported in older people [19].

**Fig. 2:**
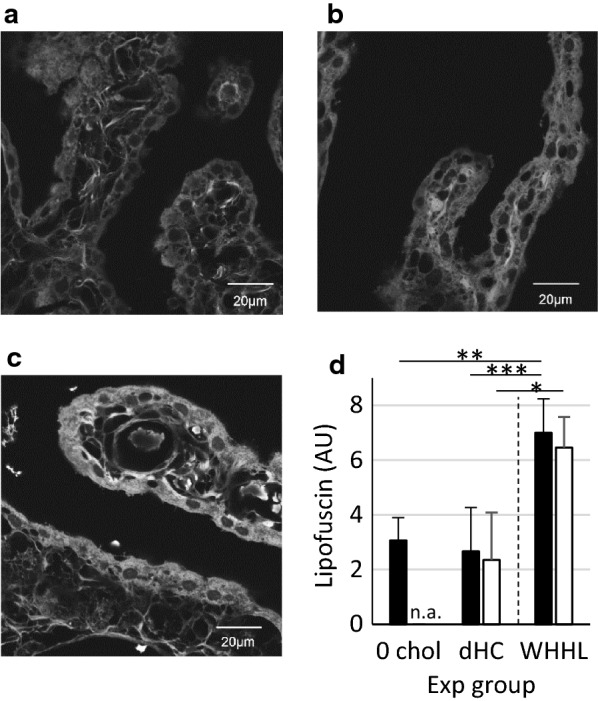
Lipofuscin levels in CPECs increase in rabbits exposed to cholesterol for longer periods. Representative images of lipofuscin in the LV CPECs from **a** 0 and **b** 16 weeks of a cholesterol diet (dHC) and **c** 32-week-old WHHL rabbits. White signals within the cytoplasm of CPECs are lipofuscin. White pixels over a threshold were measured and plotted in **d**. Bar graphs at the left of the dashed line denote Japanese White rabbits, whereas the bar at the right denotes WHHL rabbits. The number of rabbits used in this analysis is 0 chol (n = 3), dHC (n = 5) and WHHL (n = 7) for CPEC without foam cells (black bars) and 0 chol (n.a.), dHC (n = 2) and WHHL (n = 5) for CPEC with foam (white bars). Data are the mean ± standard deviation (arbitrary unit, AU), *p < 0.05, **p < 0.01 and ***p < 0.001. Scale bar = 20 μm

The area of the lipofuscin-positive signal within CPECs was measured for CPECs adjacent to or without FCs/cholesterol crystals (Fig. 2). For CPECs without FC association, WHHL rabbits had a significantly increased amount of lipofuscin compared with that in control rabbits or dHC rabbits. Likewise, CPECs adjacent to FCs had an increased amount of lipofuscin in WHHL than in dHC groups. These results suggested that excess cholesterol from the circulation and FCs induced senescence-like changes in CPECs if the duration and concentration of cholesterol exposure was longer and greater, respectively.

### AQP-1 expression

**Fig. 3:**
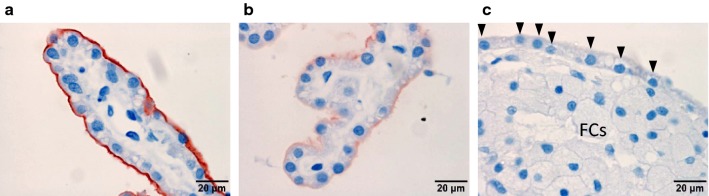
AQP1 expression at the apical membrane of CPECs diminishes in foam cell-adjacent CPECs. Representative immunohistochemistry images of AQP1 in the LV choroid plexus from **a** 0 (n = 4) and **b** 16 weeks of a cholesterol diet (n = 6) and **c** 32-week-old WHHL (n = 5) rabbits. CPEC nuclei in contact with foam cells (FCs) are marked with arrowheads. Scale bars = 20 μm

To assess functional impairment of CPECs, the expression of AQP1, one of the major molecules involved in CSF production in the CP, was visualized by immunohistochemistry. Normal rabbits without cholesterol exposure exhibited clear and strong expression of AQP1 at the apical (CSF) side of CPECs, where AQP1 has a major role in CSF production (Fig. 3a). The similar examples are shown in Additional file 1a, d, e, which show clear AQP1 positivity. In comparison, dHC shows weaker AQP1 positivity at apical side of CPECs (Fig. 3b and Additional file 1F, G, H). Also, WHHL rabbits, all show weaker expression AQP1 in CPEC without contacting foam cells (Additional file 2C, F, I, L, N). Please notice the absence of foam cells in these CP areas. This suggests that CPEC AQP1 expression is reduced in a cholesterol-exposed condition without contacting foam cells. Thus, this reduction in AQP1 expression seems to be independent of FC accumulation or starts before FC accumulation. This may suggest an effect of cholesterol or other plasma factors from the circulation. Furthermore, CPECs in contact with FCs presented diminished or virtually no expression of AQP1 (Fig. 3c; Additional file 2B, E, H, K, arrowheads) suggesting that the effect of FCs may enhance AQP1 reduction in CPEC.

### RAM11 immunoreactivity

**Fig. 4:**
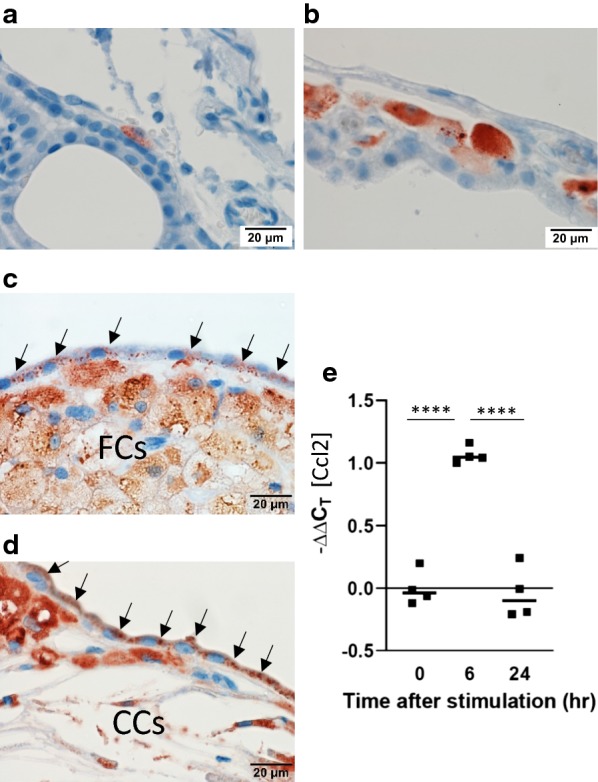
RAM11 immunoreactivity becomes positive in CPECs in rabbits exposed to cholesterol for a longer time. **a** A resident RAM11-positive macrophage is shown in the CP of a standard chow-fed rabbit. The cell is at the apical side of the CPEC. Representative of four standard chow-diet rabbits. **b** In 16-week cholesterol-fed rabbits, RAM11-positive macrophages started to accumulate within the stromal region of the CP. The number of rabbits observed is six. **c** Representative of 32-week-old WHHL (32-week cholesterol exposed) rabbits, RAM11-positive foam cells (FCs) within the CP are seen and CPECs adjacent to FCs become RAM11-positive (arrows). **d** In a region of the CP where cholesterol crystals (CCs) are formed, the punctate RAM11 immunoreactivity (arrows) of CPEC is denser. **c**, **d** are representative images of seven WHHL rabbits. Scale bars = 20 μm. CPs from LVs are shown. **e** Expression of Ccl2 mRNA from pig’s CPECs determined by qRT-PCR. The interleaved bars in the scatter plot show the median values (n = 4). Differences between multiple groups were analyzed with one-way ANOVA, followed by Tukey’s multiple comparison post hoc test. ****p < 0.0001

We aimed to confirm the origin of FCs as macrophages. Hence, localization of the rabbit-specific macrophage marker RAM11 was determined. When there was no cholesterol exposure, RAM11 immunohistochemistry revealed occasional residential macrophages attached to the apical surface of CPECs (Fig. 4a). This observation is in agreement with reports stating that such residential cells are Kolmer cells, which are scavengers of CSF [37, 38]. In dHC rabbits, infiltrating RAM11-positive macrophages in the stromal space of the CP were observed (Fig. 4b). At this point, their morphology was that of macrophages. In WHHL rabbits (which had longer exposure to cholesterol than dHC rabbits), FCs (which are thought to be cholesterol-laden macrophages) appeared as RAM11-positive cells (Fig. 4c, FCs). Contrary to our aim, we were surprised to find that CPECs in contact with FCs appeared to have RAM11-positive punctate signals in their cytoplasm (Fig. 4c, arrows). Furthermore, CPECs that surrounded cholesterol crystals developed stronger and denser RAM11-positive signals (Fig. 4d).

As a potential mechanism of the CP pathology described above, we hypothesized that the epithelium of the CP may express cytokines to induce FC accumulation in response to lipoprotein (cholesterol) insults during hypercholesterolemia. To test this hypothesis, a primary culture of CPECs from pigs was prepared on Transwell inserts. Then, the latter were stimulated with 50% fetal bovine serum (which contains lipoproteins) in culture medium added to the bottom chamber (basal side opposed to apical/CSF side). Expression of various cytokine genes in the primary culture was investigated by real-time PCR. Among them, the macrophage chemoattractant *Ccl2* showed significant induction 6 h after stimulation (Fig. 4e). Other factors tested, Cx3cl1, Mif and Vegfb, did not show any changes. This observation suggested that a cholesterol insult from the circulation stimulated CPECs to initiate macrophage infiltration to the CP stroma.

## Discussion

We showed that signs of damage were evident in the CPECs of hyperlipidemic rabbits. Consistent with a report by Chen and coworkers [27], CP stroma accumulated FCs which are macrophages with cholesterol deposits after scavenging lipoproteins from plasma. The LVs seemed predominant site for the CP to develop FC mass. For example, in WHHL 32 w animals, while all LVs had FC mass in the CP, but only half of animals had FCs in 3V CP.

In the dHC model, a normal rabbit fed a 0.3%-cholesterol diet typically exhibits an increase in total cholesterol (TC) in plasma. That is, by 2 weeks, TC in plasma reaches 200 mg/dL, and the value increases gradually to 400 mg/dL by 6 weeks, and to 800 mg/dL by 8–10 weeks. The TC level is maintained at ~ 800 mg/dL until 16 weeks [33, 34]. The second model used in this study, WHHL rabbit, has a 12-nucleotide deletion mutation in *LDLR* that causes the LDLR to reach the cell surface very slowly, the LDLR in WHHL rabbits is functionally negative [39]. Very-low-density lipoprotein remnants and LDL are not taken up by LDLR-defective hepatocytes in the liver, so WHHL rabbits are exposed continuously to high levels of plasma cholesterol in the form of lipoproteins (e.g., LDL) throughout life. Typically, the plasma level of TC is maintained at ~ 1000 mg/dL up to 32 weeks of age [26, 40]. While a 0.3%-cholesterol diet increases the plasma level of cholesterol gradually in normal rabbits, WHHL rabbits maintain their high plasma level of cholesterol during 32 weeks. Thus, the cholesterol that rabbits were exposed to was not just longer in 32-week-old WHHL rabbits compared with 16-weeks of a cholesterol diet in normal rabbits (dHC), it was also at a greater concentration in 32-week-old WHHL rabbits. Indeed, FC accumulation was seen more frequently in 32-week-old WHHL rabbits than that in dHC, although their age was identical (32 weeks; Fig. 1). Triglyceride (TG) also increases in WHHL that induces TG-rich lipoproteins such as very low density lipoprotein (VLDL) [40]. In fact, a VLDL metabolite, βVLDL is predominant lipoprotein in WHHL [41] As macrophages uptake βVLDL to accumulate excess esterified cholesterol and become FCs [42] an increase in TG may be an additional reason that WHHL produce FC mass more than dHC group. Also, cellular intake of excess triglyceride may enhance oxidization of unsaturated fatty acid to increase lipofuscin.

The CPEC height was reduced when CPECs were adjacent to FCs, and that height difference increased in WHHL rabbits (which had longer exposure to cholesterol) than dHC rabbits (Fig. 1). A reduction in CPEC height has been reported in cognitively normal elderly humans compared with their younger counterparts [19]. We also observed that prolonged exposure to cholesterol induced an increase in lipofuscin levels within CPECs (Fig. 2). Moreover, we showed that expression of AQP1 decreased at the apical membrane of CPECs in cholesterol-exposed rabbits, and diminished further in CPECs adjacent to FCs (Fig. 3). A reduction in the rate of CSF production occurs in humans with age [43]. It has been reported that the AQP1 level decreases at the apical side of CPECs in aged rats [44, 45]. Taken together, these data suggest that cholesterol (or cholesterol-laden FCs) influence CPECs to age prematurely and reduce CPEC functions.

CPECs in contact with FCs became immunoreactive to RAM11 (Fig. 4). The RAM11 monoclonal antibody was established using peritoneal macrophage lysates in rabbits as immunogens, so it is specific to rabbit macrophages [46]. The target molecule that reacts to RAM11 antibody has not been determined, but its granular staining pattern suggests that it may be enriched in secretory vesicles. Nevertheless, it has been reported that the stratified squamous epithelium in the skin, oral mucosa and esophagus are RAM11-positive [47]. Our results suggest that cholesterol exposure and/or contact with FCs altered CPEC characteristics by inducing expression of RAM11 immunoreactive molecules or by allowing the transfer of RAM11 from FCs to CPECs.

When CPEC primary culture was stimulated with serum (cholesterol/lipoprotein), it provided a quick response of chemokine *Ccl2* mRNA expression, which attracts monocytes/macrophages to the site (Fig. 4e). Scholars have reported that CPECs express and secrete CCL2 when various stimuli recruit monocytes to the CP [48–50]. Our data suggest that cholesterol insults from the circulation to the CP act as triggers to induce macrophage accumulation which, in turn, ensures FC formation at this site. In human, CSF total cholesterol is about 0.5–0.6 mg/dL, whereas normal serum total cholesterol is about 200 mg/dL, making CSF cholesterol concentration 1/300 of the serum total cholesterol [51–53] Also, CPEC has cholesterol efflux function utilizing membrane transporters such as ATP-binding cassette transporter A1 (ABCA1) and ABCG1 to transfer cholesterol from the CP to CSF, making flow of cholesterol from the CP to CSF, rather than CSF to the CP [11, 54]. Thus, it is reasonable to predict excess cholesterol within the CP of hyperlipidemic rabbits is originated from the circulation to affect CPECs.

In addition to dyslipidemia and atherosclerosis models, hyperlipidemic rabbits (such as cholesterol-fed rabbits and WHHL rabbits) have been shown to be AD models. Several research teams have described that a 2%-cholesterol diet for 8 weeks [22] and 1%-cholesterol diet for 28 weeks in wild-type rabbits [55] or male WHHL rabbits aged 48 weeks [24] display the features of AD. Such features include amyloid-beta plaques and neurofibrillary tangles in the brain as well as memory/learning dysfunctions [22, 24, 55, 56]. On the other hand, a significant reduction in the CPEC height of AD patients compared with that in cognitively normal older people has been reported [19, 57] and that lipofuscin levels within CPECs increase more in AD patients than in older healthy individuals [19]. We observed similar changes in CPEC height and lipofuscin accumulation in cholesterol-exposed rabbits in conditions milder than the AD rabbit models stated above. Therefore, our data may reflect earlier changes in CPECs than those induced in the AD models described above. In the future study, quantitation of proteins, cholesterol and oxidation of the CP, CSF and brain should be performed to elucidate the mechanism.

## Conclusions

We investigated pathologic changes in the CP of hypercholesterolemic rabbits. We found marked abnormalities in the CP epithelium as characterized by reductions in CPEC height, an increase in lipofuscin levels, and reduced expression of AQP-1. Moreover, the macrophage-specific antigen RAM11 was detected within the CPECs of FC-accumulated areas. These data demonstrate that cholesterol insults from the circulation induce CPEC deterioration, which is accelerated by FCs accumulating in the stroma of the CP.

## Supplementary information

**Additional file 1.** AQP-1 immunohistochemical (IHC) staining of controls and dHC. A) Working concentration of rabbit polyclonal anti-AQP-1 antibody (0.5 μg/mL), B) normal rabbit IgG (0.5 μg/mL) control or C) PBS (primary antibody-omitted control) were used on CP sections from a rabbit fed a 0%-cholesterol diet. D) AQP-1 IHC staining on a different rabbit consuming a 0%-cholesterol diet than the rabbit in (A). Inset (e) is shown in a magnified field in (E). Apical side of CPECs are clearly AQP-1-positive. AQP-1 IHC staining from a dHC rabbit (F) with inset (g), which is magnified in (G). AQP-1 IHC staining in the CP (H) of another dHC rabbit. The AQP-1 stain of apical CPECs is weaker than that of 0%-cholesterol diet-fed rabbits. The LV CPs are shown.

**Additional file 2.** AQP1 IHC appearance from WHHL 32w rabbits (n = 5). AQP1 immunoreaction (red) is observed in rabbit 1 (A-C), 2 (D-F), 3 (G-I), 4 (J-L) and 5 (M–N). Insets (b and c) in (A) are shown in (B) and (C), respectively in higher magnification. Insets (e and f) in (D) are shown in (E) and (F), respectively in higher magnification. Insets (h and i) in (G) are shown in (H) and (I), respectively in higher magnification. Inset (k) in (J) is shown in (K) in higher magnification. (L) shows another part of CP in the LV from rabbit 4. Inset (n) in (M) is shown in (N) in higher magnification. Scale bars in A, D, G, J, L and M are 200 μm, whereas bars in B, C, E, F, H I, K and N are 50 μm. Regions of CPEC with foam cells, where AQP1 immunoreactivity is diminished are marked with arrowheads. The LV CPs are shown.

## Data Availability

The datasets used and analyzed during the current study are available for the corresponding author on reasonable request.

## Abbreviations

CP: Choroid plexus
CSF: Cerebrospinal fluid
CPECs: Choroid plexus epithelial cells
WHHL: Watanabe hereditary hyperlipidemic
AQP-1: Aquaporin-1
AD: Alzheimer’s disease
RoI: Region of interest
*Ccl2*: C-C chemokine ligand 2
LDLR: Low-density lipoprotein receptor
TC: Total cholesterol
FCs: Foam cells
