# Supplementary figures and images for: Hypercholesterolemia negatively influences morphology and molecular markers of epithelial cells within the choroid plexus in rabbits
Source: Fluids Barriers CNS. 2020 Feb 4;17:13. doi: 10.1186/s12987-020-0175-0 (PMC7001221; doi:10.1186/s12987-020-0175-0)

## Slide 1
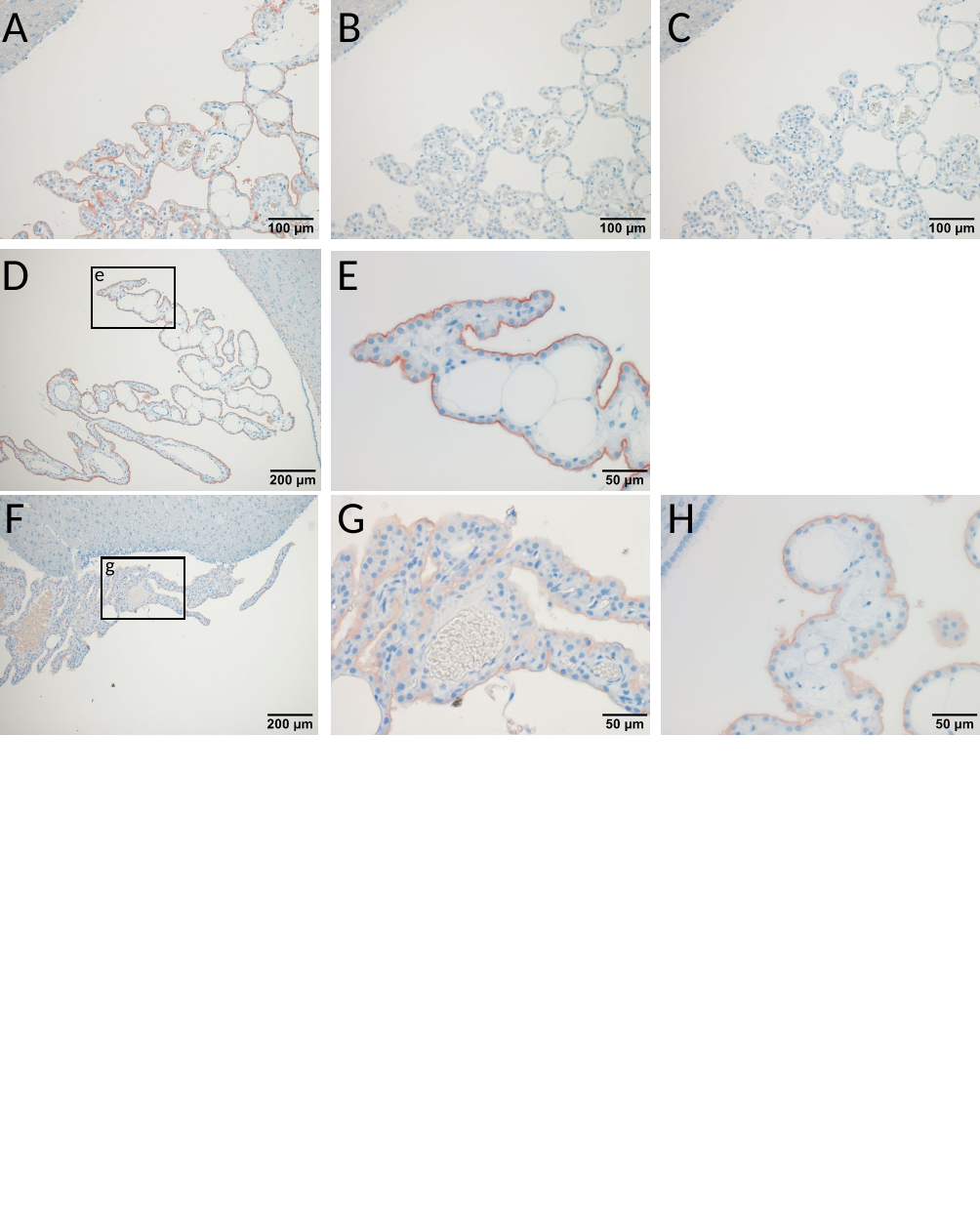

A
B
C
D
E
e
G
H
F
g

Supplement: Supplementary file 1 — Additional file 1. AQP-1 immunohistochemical (IHC) staining of controls and dHC. A) Working concentration of rabbit polyclonal anti-AQP-1 antibody (0.5 μg/mL), B) normal rabbit IgG (0.5 μg/mL) control or C) PBS (primary antibody-omitted control) were used on CP sections from a rabbit fed a 0%-cholesterol diet. D) AQP-1 IHC staining on a different rabbit consuming a 0%-cholesterol diet than the rabbit in (A). Inset (e) is shown in a magnified field in (E). Apical side of CPECs are clearly AQP-1-positive. AQP-1 IHC staining from a dHC rabbit (F) with inset (g), which is magnified in (G). AQP-1 IHC staining in the CP (H) of another dHC rabbit. The AQP-1 stain of apical CPECs is weaker than that of 0%-cholesterol diet-fed rabbits. The LV CPs are shown. [file 12987_2020_175_MOESM1_ESM.pptx]

## Slide 1
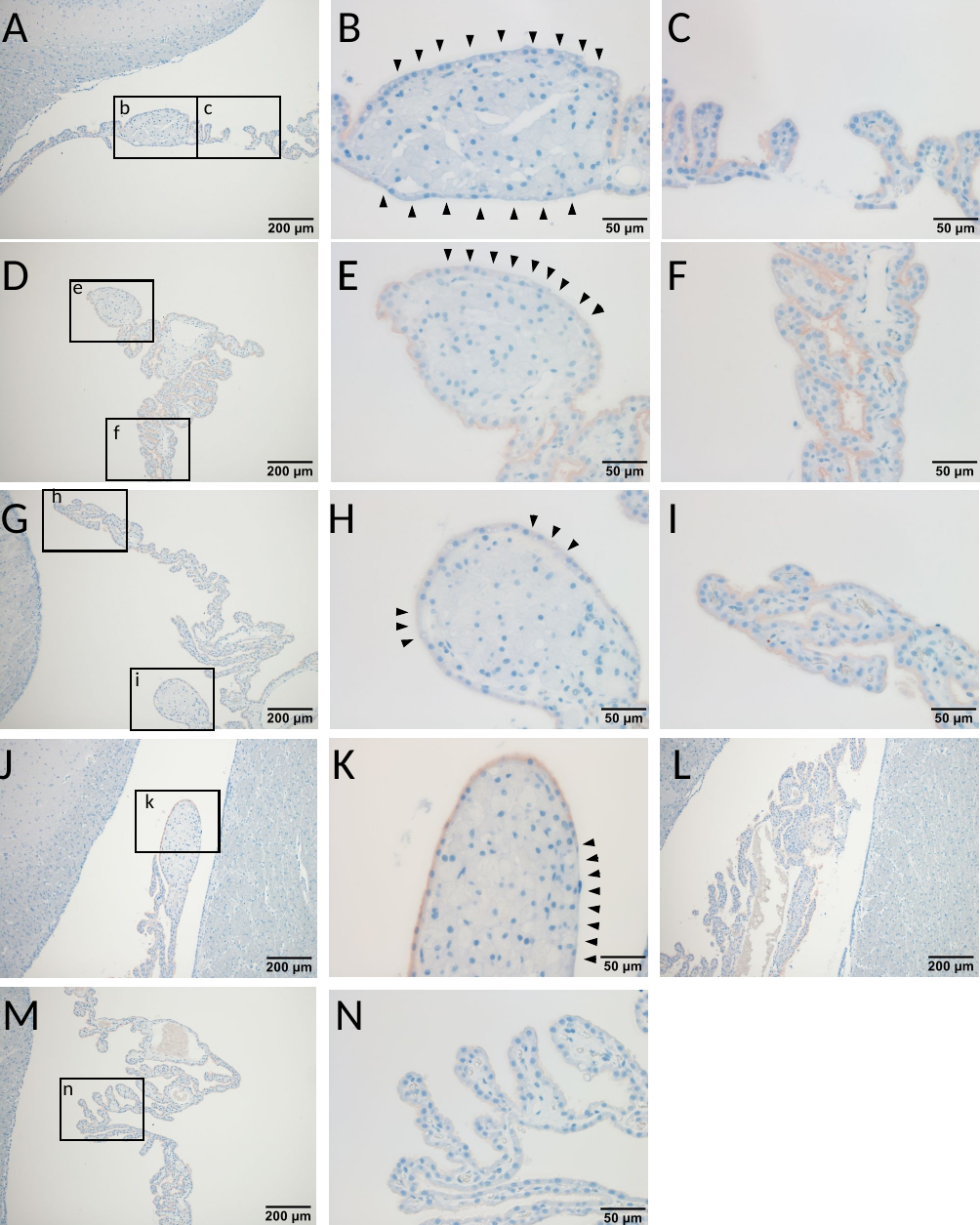

A
B
C
c
b
D
E
E
F
e
f
h
G
H
I
i
J
K
L
k
M
N
n

Supplement: Supplementary file 2 — Additional file 2. AQP1 IHC appearance from WHHL 32w rabbits (n = 5). AQP1 immunoreaction (red) is observed in rabbit 1 (A-C), 2 (D-F), 3 (G-I), 4 (J-L) and 5 (M–N). Insets (b and c) in (A) are shown in (B) and (C), respectively in higher magnification. Insets (e and f) in (D) are shown in (E) and (F), respectively in higher magnification. Insets (h and i) in (G) are shown in (H) and (I), respectively in higher magnification. Inset (k) in (J) is shown in (K) in higher magnification. (L) shows another part of CP in the LV from rabbit 4. Inset (n) in (M) is shown in (N) in higher magnification. Scale bars in A, D, G, J, L and M are 200 μm, whereas bars in B, C, E, F, H I, K and N are 50 μm. Regions of CPEC with foam cells, where AQP1 immunoreactivity is diminished are marked with arrowheads. The LV CPs are shown. [file 12987_2020_175_MOESM2_ESM.pptx]
